# Validation of an Optimised Method for Quantitative Detection of Hepatitis E Virus in Pork Sausage

**DOI:** 10.1007/s12560-025-09645-3

**Published:** 2025-06-02

**Authors:** Sofia Persson, Ramia Molin, Ronnie Eriksson, Moa Lavander, Frederik Widén, Patrik Ellström, Magnus Simonsson

**Affiliations:** 1 European Union Reference Laboratory for Foodborne Viruses, Swedish Food Agency, Dag Hammarskjölds väg 56 A, 752 37 Uppsala, Sweden; 2 Department of Medical Sciences, Zoonosis Science Centre, Uppsala University, Husargatan 3, 752 37 Uppsala, Sweden; 3 Department of Microbiology, Swedish Veterinary Institute, Ulls väg 2B, 751 89 Uppsala, Uppsala, Sweden

**Keywords:** HEV, Real-time PCR, Digital PCR, Foodborne virus, Validation, Method characterisation

## Abstract

Hepatitis E virus (HEV) infection is an emerging zoonosis that can be transmitted to humans through the consumption of raw or undercooked pork products. Several methods have been described to detect the virus in food, but there are few data on qualitative and quantitative performance characteristics. In this study, we have developed an optimised method for quantitative detection of HEV in pork sausage based on a combination of previously published protocols. The method utilises sample disruption and phase separation with tri-reagent and 1-bromo-3-chloropropane followed by RNA concentration with isopropanol precipitation. We validated the method for use on reverse transcription quantitative real-time PCR (RT-qPCR) and reverse transcription droplet digital (RT-ddPCR). The 95% limit of detection and limit of quantification was 200 copies/g for both RT-qPCR and RT-ddPCR. RT-ddPCR technology has previously shown promise as a more precise alternative to RT-qPCR. However, we found no evidence for improved performance using RT-ddPCR instead of RT-qPCR for this method. Additionally, we further verified the performance of the HEV RT-PCR assay using the WHO International Standard and Reference Panel for HEV RNA. Finally, we assessed different combinations of RNA concentration protocols and RT-PCR detection strategies. This showed that isopropanol precipitation of viral RNA was at least twice as efficient as magnetic silica bead-based extraction when an inhibitor-tolerant RT-qPCR detection strategy was used. In summary, we present an efficient and well-characterised method for quantitative detection of HEV in pork sausage. Such methods are valuable to provide high-quality data for risk assessments and food monitoring.

**Supplementary Information:**

The online version contains supplementary material available at 10.1007/s12560-025-09645-3.

## Introduction

Hepatitis E virus (HEV) is an emerging pathogen of increasing public health concern. Recent research suggests that approximately 12% of the global population have been infected with the virus (Li et al., 2020). While most HEV infections are asymptomatic or manifest as mild disease, about 5–30% of infected individuals develop acute icteric hepatitis. The incubation period is two to six weeks and the disease is typically self-limiting. However, patients with underlying liver disease are at risk of developing life-threatening acute liver failure. Furthermore, in severely immunocompromised individuals, HEV infection can be prolonged or become chronic (Lhomme et al., 2020). Emerging evidence also points to a causal relationship between HEV infection and various neurological conditions, such as neuralgic amyotrophy and Guillain–Barré syndrome, as well as renal manifestations and kidney damage (Dalton et al., 2016; Kamar et al., 2011; Ripellino et al., 2020; Webb & Dalton, 2020).

Human HEV strains are found within five (genotype 1–4 and 7) out of the eight known genotypes within the *Orthohepevirus* A species of the family *Hepeviridae* (Purdy et al., 2017). Genotypes 1 and 2 are considered human-specific, while genotypes 3 and 4 are zoonotic with domestic pigs and wild boars serving as primary reservoirs. Genotype 3 is widespread in Europe, the Americas, and Asia, while genotype 4 is mainly found in Northeast Asia (Lhomme et al., 2020; Smith & Simmonds, 2018). The HEV genome consists of a single-stranded, positive-sense RNA molecule of 6.4 to 7.2 kilobases, containing three partially overlapping open reading frames (ORFs) (Purdy et al., 2017). The virion is non-enveloped in faeces and quasi-enveloped in blood (Yin et al., 2016).

One of the most common routes of transmission of zoonotic HEV to humans is the consumption of raw or inadequately cooked pork products, particularly liver and cold-smoked, fermented or air-dried sausages (Hazards et al., 2017; Treagus et al., 2021). Various methods for detecting the virus in pork products are described [for instance; (Althof et al., 2019; Bartolo et al., 2012; Colson et al., 2010; Hennechart-Collette et al., 2019; Martin-Latil et al., 2014; Mykytczuk et al., 2017; Szabo et al., 2015)] and a standardisation process for a qualitative method was initiated by the International Organisation for Standardisation in 2022 (ISO; ISO/TC 34/SC 9/WG31 Hepatitis E virus). For two other common foodborne viruses, hepatitis A virus (HAV) and norovirus, a qualitative and quantitative ISO method already exists for the most relevant food types [ISO 15216–1 and −2 (I.S.O., 2017, 2019)]. In the validation of ISO 15216–1:2017, performance characteristics such as limit of detection (LOD) and limit of quantification (LOQ) were determined on different food types by testing 54 food samples contaminated with a dilution series of the target viruses (Lowther et al., 2019). To the best of our knowledge, no quantitative method for detecting HEV in food has been characterised to the same extent. Information on quantitative aspects of method performance is desirable to provide robust data for risk assessments and for official control purposes.

In this study, we developed and validated an optimised method for the extraction of HEV from a pork product by integrating two published protocols (Mykytczuk et al., 2017; Szabo et al., 2015). For quantitative detection of viral RNA, we employed both reverse transcription (RT) quantitative real-time PCR (RT-qPCR) and droplet digital RT-PCR (RT-ddPCR). The LOD and LOQ were determined by using a strategy similar to that used for characterising ISO 15216–1:2017 (Lowther et al., 2019). To ensure the reliability of the selected HEV RT-PCR assay, we conducted an in silico inclusivity analysis using the GenBank database and a laboratory evaluation using the WHO International Standard (S. A. Baylis et al., 2013) and the WHO International Reference Panel (Baylis et al., 2015) for HEV RNA quantification. Additionally, we performed a comparative evaluation of different RNA purification methods and RT-PCR detection strategies. This evaluation included assessments of HEV concentrations obtained, the degree of RT-PCR inhibition, and the total RNA concentration yielded by each RNA purification method.

## Materials and Methods

### Preparation of HEV Spiking Material

A liver tissue sample (approximately 0.5 g) from a wild boar, naturally infected with HEV-3, kindly provided by the Swedish Veterinary Institute, was used as spiking material throughout the study. The material was carefully homogenised using sterile scalpels. The homogenate was dissolved by repeated pipetting in 4 ml refrigerated, sterile RNA storage solution (Invitrogen). The dissolved homogenate was centrifuged at 10,000 × g at 4 °C for 10 min to remove any larger debris. The supernatant was collected and stored in single-use aliquots at − 70 °C.

### Protocols for Analysis of HEV in Sausage

#### Isolation of Viral RNA

To isolate viral RNA from sausage samples, 4.0 g of finely chopped sausage was placed in an IKA ULTRA-TURRAX disperser tube (DT-20) (Sigma-Aldrich). Then, 10 µl of mengovirus positive process control (consisting of 1/10 diluted cell culture supernatant of mengovirus strain vMCO, CECT 100000) was pipetted onto the sample, followed by the addition of 12 ml of tri-reagent solution (Sigma-Aldrich). To homogenise the sample, the disperser tube was connected to an IKA ULTRA-TURRAX tube drive (Sigma-Aldrich) and run at 2000 rpm for 2 min. The homogenate was transferred to a 50 ml Falcon tube and centrifuged at 10,000 × g for 20 min at 4 °C. The supernatant was transferred to a clean 50 ml Falcon tube, to which 1.2 ml of 1-bromo-2-chloropropane (Sigma-Aldrich) was added to promote separation between the protein, DNA, and RNA phases. The sample was vortexed for 15 s and incubated for 10 min at room temperature, followed by centrifugation at 10,000 × g for 15 min at 4 °C. The aqueous phase (with RNA) was transferred to a 1.5 ml Eppendorf tube. The total volume of the aqueous phase was noted and the sample was either processed directly or stored at -70 °C until RNA concentration. A negative process control (NPC) consisting of 4 ml phosphate buffered saline (PBS) was analysed in parallel with the samples in each round. 

#### RNA Concentration and Purification

Two RNA concentration methods were used in this study: isopropanol precipitation (Mykytczuk et al., 2017) and guanidine thiocyanate-magnetic silica bead extraction (Boom et al. 1990).

Isopropanol precipitation was performed by placing 1000 µl of the sample in a 1.5 ml Eppendorf tube along with 500 µl of molecular grade isopropanol (Thermo Fisher Scientific). The tube was inverted 5–10 times, incubated at room temperature for 10 min, and then centrifuged at 12,000 × g at 4 °C for 10 min. The supernatant was aspirated by vacuum, using a VACUBOY vacuum aspirator (Integra). The RNA pellet was washed by adding 1000 µl of 75% molecular grade ethanol (Thermo Fisher Scientific). The sample was vortexed for 5 s and centrifuged at 7500 × g for 5 min at 4 °C. The supernatant was again aspirated using vacuum and the RNA pellet was dried with the lid open for 10 min at room temperature. The pellet was dissolved by adding 100 µl of nuclease-free water and the sample was placed on a 60 °C heat block for 5 min, while shaking at 1400 rpm. A boil lysate was used as a positive extraction control. It was prepared by adding 10 µl of mengovirus and 10 µl of HEV spike material to 80 µl of PBS in a 1.5 ml Eppendorf tube and heating at 95 °C for 5 min. 

Guanidine thiocyanate-magnetic silica bead extraction was performed using a NucliSENS miniMAG instrument and NucliSENS magnetic extraction reagents (Biomérieux), hereafter referred to as Minimag, according to the manufacturer’s instructions, with 1000 µl sample volume and 100 µl elution volume. A positive extraction control was prepared by adding 10 µl of mengovirus and 10 µl of HEV spike material to 980 µl of PBS. The positive extraction control was processed together with the samples.

#### Primers and Probes

The primers and probes used in this study are listed in Table 1. Primers were purchased from Integrated DNA Technologies and probes were purchased from Thermo Fisher Scientific.

**Table 1:** Primers and probes used in this study

Target	Application	Type	Sequence (5′–3′)	References
HEV	RT-qPCR/RT-ddPCR	Forward primer	GGTGGTTTCTGGGGTGAC	(Jothikumar et al., 2006)
HEV	RT-qPCR/RT-ddPCR	Reverse primer	AGGGGTTGGTTGGATGAATATAG	(Martin-Latil et al., 2012)
HEV	RT-qPCR/RT-ddPCR	Probe	[FAM]-TGATTCTCAGCCCTTCGC-[MGB][EQ]	(Garson et al., 2012)
Mengovirus	RT-qPCR	Forward primer	GCGGGTCCTGCCGAAAGT	(Pintó et al. 2009)
Mengovirus	RT-qPCR	Reverse primer	GAAGTAACATATAGA CAGACGCACAC	(Pintó et al. 2009)
Mengovius	RT-qPCR	Probe	[FAM]-ATCACATTACTG GCCGAAGC-[MGB][EQ]	(Pintó et al. 2009)

FAM = 6-carboxyfluorescein, MGB-EQ = minor groove binder-eclipse quencher

#### HEV DNA Quantification Standard and External Control RNA

To generate a HEV DNA quantification standard and external control (EC) RNA, the following sequence: TAATACGACTCACTATAGCCGCCGTCGTGGGCGGCGCAGCGGCGGTGCCGGCGGTGGTTTCTGGGGTGACAGGGTTGATTCTCAGCCCTTCGCCCT**GGAT**CCCCTATATTCATCCAACCAACCCCTTCGCCGCCGACGTCGTATCACAATCCGGGGCTGGAGCTCGCCCTC**AAGCTT**, was ligated into pEXA2 vector at Eurofins Genomics. The insert is based on the consensus sequence from an alignment of HEV sequences and corresponds to position 5203–5351 in the HEV reference sequence (NC_001434). The insert contains a T7 promoter at the 5′ end, a *Hin*dIII site at the 3′ end (**AAGCTT**), and a *Bam*HI site in the amplicon region to be able to distinguish from naturally occurring targets (**GGAT**CC; bold letters indicate added bases). The plasmid was linearised with *Hin*dIII (New England Biolabs) and separated by 1% agarose gel (Sigma-Aldrich) electrophoresis at 100 V for 2.5 h. The linear gel band was excised and purified with QIAquick Gel Extraction Kit (Qiagen). External control RNA was prepared using Riboprobe Combination System T7 RNA Polymerase (Promega) and then treated with DNase according to the manufacturer’s protocol. Transcripts were purified using the RNeasy MinElute Cleanup Kit (Qiagen). All transcripts were checked for DNA content less than 0.1% by qPCR with and without reverse transcription. Plasmids and transcripts were quantified using a Qubit 3.0 Fluorometer (Thermo Fisher Scientific). The material was diluted in 1 X TE buffer (Sigma-Aldrich), divided into single-use aliquots and stored at − 70 °C.

#### RT-qPCR

RT-qPCR was performed on a Roche LightCycler 96 System. Each reaction contained 500 nM forward primer, 900 nM reverse primer, and 150 nM (HEV) or 250 nM (mengovirus) probe. Reaction mixes were prepared with 20 µl of reagents and 5.0 µl of sample. Two mastermixes were used in this study: TaqPath 1-Step RT-qPCR Master Mix, CG (Mastermix A), and RNA UltraSense One-Step Quantitative RT-PCR System (Mastermix B), both from Thermo Fisher Scientific. The cycling conditions were as follows: Mastermix A: reverse transcription at 50 °C for 15 min, inactivation of reverse transcriptase/activation of DNA polymerase at 95 °C for 2 min, followed by 45 cycles of denaturation at 95 °C for 3 s, and annealing and elongation at 60 °C for 30 s; Mastermix B: reverse transcription at 55 °C for 60 min, inactivation of reverse transcriptase/activation of DNA polymerase at 95 °C for 5 min, followed by 45 cycles of denaturation at 95 °C for 15 s, annealing at 60 °C for 60, s and elongation at 65 °C for 60 s. Results were analysed using LightCycler 96 software, version 1.1 (Roche).

A standard curve for each target was included in each RT-qPCR run. The HEV quantification standard consisted of a serial dilution of plasmid DNA ranging from 500,000 to 50 copies/reaction. The mengovirus quantification standard consisted of a serial dilution of the positive extraction control, ranging from undiluted to 1/1000 diluted. At least two no template controls (NTCs) were included in each run. All samples and controls were analysed in duplicate wells.

#### RT-ddPCR

RT-ddPCR was performed on a Bio-Rad Droplet Digital PCR platform (QX200) with One-Step RT-ddPCR Advanced Kit for Probes (Bio-Rad). Each reaction contained 500 nM forward primer, 900 nM reverse primer, and 150 nM (HEV) probe. Each sample (5.5 µl template and 16.5 µl reaction mix) was first applied to a 96-well plate, with a final volume of 22 µl (20 µl + 10%). 20 µl of each sample was then transferred to DG8™ Cartridges (Bio-Rad), and 70 µl of Droplet Generation Oil for Probes was added (Bio-Rad). Droplets were generated with a QX200 Droplet Generator (Bio-Rad) for the method characterisation study and a QX200 Automated Droplet Generator (Bio-Rad) for the analysis of the WHO Standards. In both cases, 40 µl droplet suspension (containing 20 µl sample) was transferred to a 96-well plate (Eppendorf, Hamburg, Germany), and RT-PCR was performed on a T100 Thermal Cycler (Bio-Rad) with RT at 50 °C for 1 h, inactivation of the reverse transcriptase, and DNA polymerase activation at 95 °C for 10 min, followed by 50 cycles of denaturation at 95 °C for 30 s, and annealing/elongation at 60 °C for 1 min. A final enzyme deactivation step was performed at 98 °C for 10 min. Plates were transferred to the QX200 Droplet Digital PCR system (Bio-Rad) either the same day or the day after the reaction. Results were visualised in QuantaSoft Analysis Pro software version 1.0 (Bio-Rad). At least two no template controls (NTCs) were included in each run.

#### Calculation of RT-PCR Inhibition

The HEV RT-PCR inhibition was monitored by spiking a sample well with 2.0 μl of EC RNA and comparing it to a control well with 2.0 μl EC RNA in 5.0 µl nuclease-free water (Qiagen). Inhibition ($I$) was calculated as follows:

$$I =100\bullet (1-{c}_{EC RNA sample well}/{c}_{EC RNA control well} ),$$

where $c$ denotes the estimated EC RNA concentration in copies/µl in PCR. Inhibition values of 75% or less were considered acceptable (I.S.O., 2017).

#### Calculation of Viral Genomes/g Sausage and Viral Extraction Efficiency

In RT-qPCR, a sample was regarded as positive if at least one of the two replicate PCR wells had a Cq value. In RT-ddPCR, a sample was regarded as positive if at least one of the two replicate PCR wells had at least one positive droplet. The viral concentration in the sample (${c}_{sample}$) was calculated as follows:

$${c}_{sample}= \left({c}_{RT-PCR well}\bullet {v}_{RNA eluate}/{v}_{RT-PCR}\right)/{v}_{RNA extraction}\bullet {v}_{RNA phase},$$

where ${c}_{RT-PCR well}$ denotes the obtained RNA concentration in the RT-PCR well, ${v}_{RNA eluate}$ the elution volume from the RNA concentration step (in this case 0.1 ml), ${v}_{RT-PCR}$ the sample volume in RT-PCR (in this case 0.005 ml), ${v}_{RNA extraction}$ the volume taken for RNA concentration (in this case 1 ml), and ${v}_{supernatant}$ the total volume of the aqueous phase. The number of copies/g was calculated by dividing ${c}_{sample}$ with 4 (since 4 g sample was used).

Viral extraction efficiency was calculated in all samples for mengovirus and neat samples for HEV. The total number of viral copies in the positive extraction control was calculated as follows:

$${c}_{PEC}= {c}_{RT-PCR well}\bullet {v}_{ RNA eluate}/{v}_{RT-PCR},$$

where ${c}_{RT-PCR well}$ denotes the obtained RNA concentration in the RT-PCR well, ${v}_{RNA eluate}$ the elution volume from the RNA concentration step (in this case 0.1 ml), ${v}_{RT-PCR}$ the sample volume in RT-PCR (in this case 0.005 ml).

Viral extraction efficiency ($E$) was then calculated as follows:

$$E= {c}_{sample}/{c}_{PEC}\bullet 100$$

## Method Characterisation

### Overview and Experimental Design

The method characterisation study was performed by analysing sausage artificially contaminated with a two-fold dilution series of HEV spiking material, ranging from neat (undiluted) to dilution 1/256. The procedure was carried out in seven rounds, resulting in a total of 63 spiked samples (nine dilution levels with seven subsamples per dilution level). Each subsample was analysed using both RT-qPCR and RT-ddPCR. The resulting data were used to determine quality control criteria (inhibition and viral extraction efficiency) and performance characteristics (LOD, LOQ, and quantitative agreement between RT-qPCR and RT-ddPCR).

### Preparatory Steps and Artificial Contamination

Frankfurter sausage was selected as a model matrix for characterisation and comparison because it is a popular sausage that has been identified as a risk factor for HEV infection (Faber et al., 2018). The material was finely minced with scalpels and divided into individual portions of 4 g and stored at − 20 °C until use. To ensure that the material was not naturally contaminated with HEV, six non-spiked sausage samples were analysed using the isopropanol precipitation protocol, followed by RT-qPCR analysis with Mastermix A.

To find an appropriate neat contamination level, 10 µl of HEV spiking material was added to one sample of each matrix and analysed using the isopropanol precipitation protocol followed by RT-qPCR analysis with Mastermix A. The obtained concentration was noted and the material was diluted to concentrations expected to provide approximately 5000 copies/g in sausage samples. The neat spiking material was divided into single-use aliquots and stored at -70 °C until use.

### Laboratory Analysis

For each round, the previously prepared sausage samples were thawed for 20 min at room temperature. Meanwhile, a two-fold dilution series of spiking material was prepared by adding 50 µl of HEV spiking material to 50 µl PBS in 1.5 ml Eppendorf tubes. The dilution series ranged from neat to 1/256 dilution. 10 µl from each dilution level was added to the samples at the same step as where the mengovirus was added. Samples were allowed to stand at room temperature for 10 min before starting the analysis. RNA purification was performed using the isopropanol precipitation protocol. All extractions were stored at − 70 °C before RT-PCR. RT-qPCR analysis was performed with Mastermix A. Mengovirus (the positive process control virus) was only analysed with RT-qPCR.

### Determination of Performance Characteristics

Performance characteristics were determined separately for the RT-qPCR and RT-ddPCR data. The overall procedure was similar to that described for the characterisation of ISO 15216–1:2017 (Lowther et al., 2019), with minor modifications.

### Anticipated Values

Anticipated values were calculated by taking the geometric mean of the obtained results for all samples of the most concentrated (neat) dilution, followed by multiplication with the dilution factor (i.e. 1, 1/2, …, 1/256).

### Limit of Detection

LOD_50%_ and LOD_95%_ were determined by using the Excel programme PODLOD (Wilrich & Wilrich, 2009) by giving the anticipated values together with the number of positives and total samples at each dilution level as input data.

### Limit of Quantification

The determination of LOQ was based on a procedure previously described (Lowther et al., 2019). First, all anticipated and obtained concentrations from neat to the dilution level immediately below the LOD_95%_ were log_10_ transformed. Linear regression analyses were then carried out with log_10_ obtained concentration as a function of log_10_ anticipated concentration. Each regression analysis covered three dilution levels, starting from the lowest point and then moving up one dilution level at a time. The LOQ was determined as the level above which the residual SDs from the regression analyses were always below 0.5 log_10_ (a residual is the distance between an observed value and the value predicted from the regression line. The residual SD thus shows how much the data points are scattered around the regression line, and highly scattered data points indicate poor precision). The LOQ was set no lower than the LOD_95%_.

### Comparison of Precision Between RT-qPCR and RT-ddPCR

The overall precision of RT-qPCR and RT-ddPCR was determined from LOQ up to the neat dilution. For each method separately, a linear regression analysis was performed with log_10_ obtained value as a function of log_10_ anticipated value. Precision was estimated by calculating the residual SD and the variance. The precision of RT-qPCR and RT-ddPCR was compared by performing two-sided *F* tests for the residual variance ratio by using the *var.test* function in R (R, 2021) under the null hypothesis that the residual variances associated with the two methods are equal. A result was considered significant if the *p *value was ≤ 0.05.

### Quantitative Agreement Between RT-qPCR and RT-ddPCR

Quantitative agreement between RT-qPCR and RT-ddPCR was estimated on all samples that were positive with both detection methods, by using Bland and Altman’s method (Bland & Altman, 1986).

## Method Comparison

### Overview and Experimental Design

The purpose of this experiment was to compare the performance of two RNA purification protocols and three RT-PCR protocols: RT-qPCR with two different mastermixes and RT-ddPCR. The aqueous (RNA) phases from all seven 1/2 diluted samples from the method characterisation study were pooled together and split into 1 ml portions. Seven portions were concentrated with isopropanol precipitation and seven with Minimag and eluted in volumes of 100 µl. The total RNA concentration of each eluate was quantified using a Nanodrop One UV–Vis spectrophotometer (Thermo Fisher Scientific). The HEV content was quantified using RT-qPCR with mastermixes A and B and RT-ddPCR, resulting in six RNA purification/RT-PCR protocol combinations. RT-PCR inhibition was monitored in each sample.

### Data Analysis

The total (log_10_) RNA concentrations obtained from isopropanol precipitation and Minimag extraction were compared using a two-sided Wilcoxon signed-rank test. A result was considered significant if the *p *value was ≤ 0.05.

The mean HEV concentrations obtained from the different RNA purification/RT-PCR protocol combinations were studied using a two factor ANOVA. Obtained HEV concentration (log_10_ copies/RT-PCR well) was used as a response variable, and RNA purification method and RT-PCR protocol were used as fixed factors. An interaction term between RNA purification method and RT-PCR protocol was also included. The analysis was conducted using the *lm* function from base R (R, 2021) and the *Anova* function from the *car* R-package (Fox & Weisberg, 2019). Pair-wise post hoc comparisons of the different RNA purification/RT-PCR protocol combinations were performed with Tukey’s honest significant difference (HSD) test, by using the *emmeans* R-package (Lenth et al., 2021).

### In silico* Inclusivity Analysis of HEV Primers and Probe*

Inclusivity, the ability of the assay to detect target sequences of interest (Hedman et al., 2018) was assessed by measuring the proportion of matching sequences within the intended primer and probe binding regions, using functions from the rprimer R-package (Persson et al., 2022). HEV target sequences were collected by searching for ‘E’[porgn:__txid1678143] (the taxid of *Orthohepevirus* A) on the nucleotide database of NCBI GenBank (accessed on October 2022), and filtering for sequence length > 6000 and < 8000. All sequences (*n* = 878) were downloaded and analysed using the Hepatitis E virus genotyping tool version 0.1 (RIVM 2021). Thirty-six sequences that were not classified as *Orthohepevirus* A and nine sequences with long segments of consecutive ‘N’ were removed from the dataset. The remaining 834 sequences were aligned using the online resource mafft version 7 (Katoh et al., 2019), with the reference sequence of HEV (NC_001434) as the reference alignment.

### Evaluation Using the WHO International Standard and Reference Panel

The selected HEV RT-PCR assay was evaluated using the WHO International Standard, which consists of HEV-positive serum quantified at 250,000 international units (Baylis et al., 2013), and the WHO Reference Panel, which includes 11 HEV-positive serum and stool samples representing various genotypes and sub-genotypes (Baylis et al., 2015). Lyophilised standards were purchased from the Paul-Ehrlich-Institut, Germany, with product codes #6329/10 and #8578/13, respectively. Upon arrival, the materials were carefully reconstituted in 500 µl of nuclease-free water. Subsequently, 160–200 µl of the reconstituted standards were extracted using an EMAG instrument (bioMérieux) following the manufacturer’s instructions, with an elution volume of 60 µl. The extracted material was then quantified using RT-ddPCR.

### Figures

The figures were generated using the ggplot2 R-package (Wickham, 2011) and patchwork R-package (Pedersen, 2020).

## Results

### Method Characterisation

To characterise the method for quantitative detection of HEV in sausage, samples from sausage spiked with a two-fold dilution series of HEV were analysed, with seven samples per dilution level, ranging from undiluted (neat) to 1/256 diluted. In addition, six non-spiked sausage samples were analysed to ensure that they were not naturally contaminated with HEV.

### Non-spiked Sausage Samples and Negative Controls

All six non-spiked samples were negative by RT-qPCR. However, with RT-ddPCR, one positive droplet was observed in one of the samples. In addition, all 7 NPCs and all 14 NTCs in the method characterisation study were negative in RT-qPCR. In the RT-ddPCR, 1 of 7 NPCs and 1 of 29 NTCs had 1 positive droplet.

### RT-PCR Inhibition

All samples met the acceptance criterion of a maximum inhibition of 75%. The average inhibition for the HEV detection assay was 15% with RT-qPCR (SD: 10%; range: − 38% to 31%) and 15% with RT-ddPCR (SD: 18%, range: − 22% to 61%).

### Viral Extraction Efficiency

Viral extraction efficiency was measured on RT-qPCR for both HEV and the mengovirus positive process control by using a boil lysate as a reference. The average extraction efficiency was 137% (SD: 39%; range: 87% to 187%) for HEV and 118% (SD: 36%; range: 3% to 192%) for mengovirus.

### Limit of Detection and Limit of Quantification

**Fig. 1:**
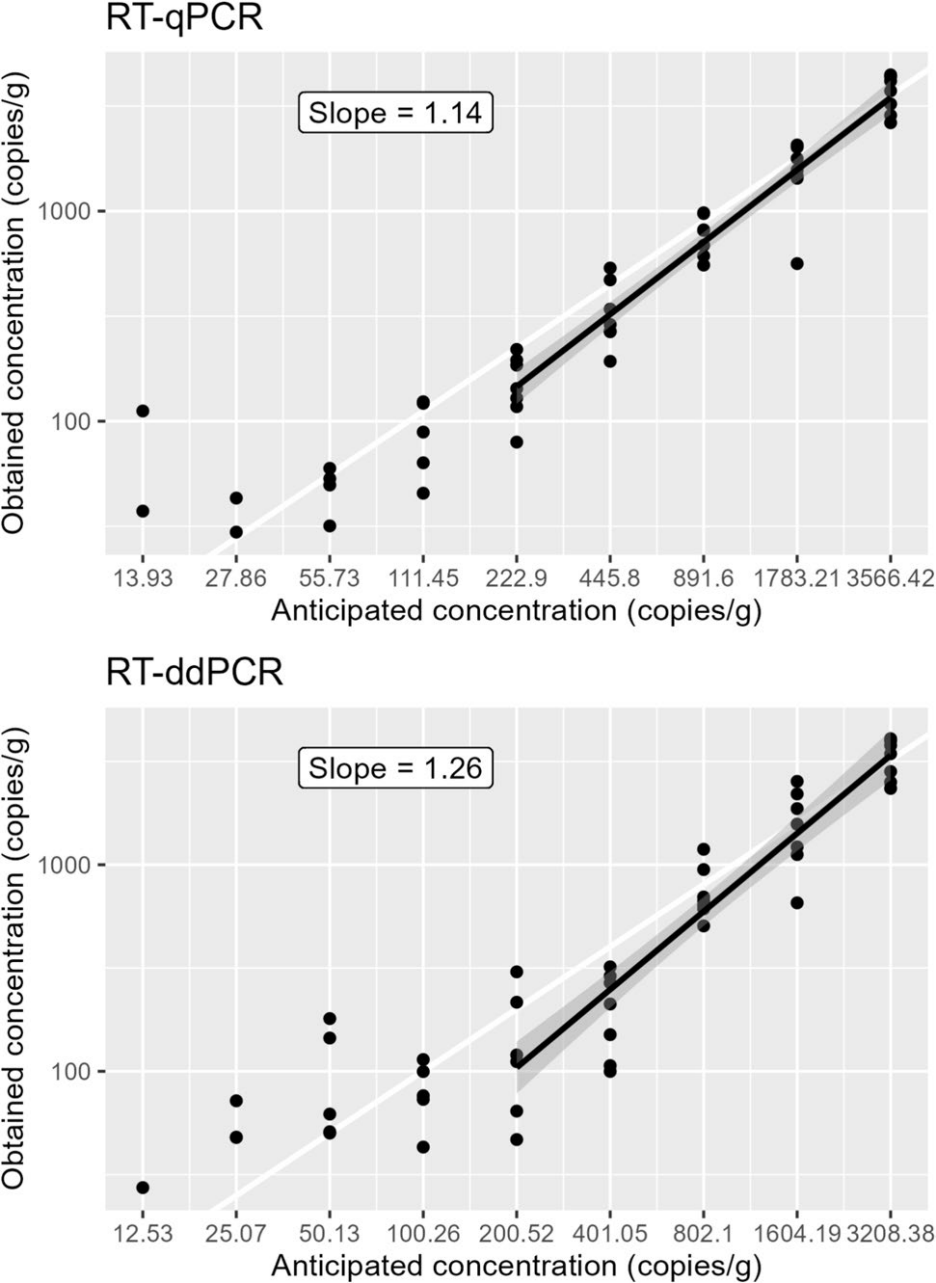
Results from the method characterisation study, obtained versus anticipated values for RT-qPCR and RT-ddPCR. Dots represent observed values. The thick black line represents fitted values from linear regression covering the data points with an anticipated concentration higher than LOQ and the shaded areas represent a pointwise 95% confidence intervals of the fitted values. The thick white line is the line of equivalence, i.e. where the anticipated concentration equals the obtained concentration

Limit of detection and limit of quantification were determined on the basis of all samples in the method characterisation study. An overview of the results is given in Fig. 1, the number of positive samples at each dilution level and coefficient of variation (% CV) of the measured concentrations are given in Table 2 and LOD_95%_, LOD_50%_, and LOQ are given in Table 3. Note that the LOD and LOQ estimates are based on anticipated concentration and are therefore not directly comparable between RT-qPCR and RT-ddPCR. When compensating for the small difference in how the two methods quantify (by setting the same anticipated concentration for both methods), the estimated LOD_95%_ and LOD_50%_ were 11% higher with RT-ddPCR than with RT-qPCR.

**Table 2:** Summarised results from the method characterisation study

	RT-qPCR				RT-ddPCR			
Dilution level	Anticipated conc. (copies/g)	Total number of observations	Number of positive observations	% CV	Anticipated conc. (copies/g)	Total number of observations	Number of positive observations	% CV
Neat	3600	7	7	20	3200	7	7	22
½	1800	7	7	32	1600	7	7	41
¼	890	7	7	22	800	7	7	31
1/8	450	7	7	36	400	7	7	43
1/16	220	7	7	32	200	7	6	68
1/32	110	7	5	39	100	7	5	34
1/64	56	7	4	25	50	7	5	62
1/128	28	7	2	26	25	7	3	25
1/256	14	7	2	70	13	7	1	–

**Table 3:** 50% and 95% limit of detection and limit of quantification of HEV in sausage, anticipated concentration, expressed as copies/g

Detection method	LOD_50%_ (95% CI)	LOD_95%_ (95%CI)	LOQ
RT-qPCR	47 (28–78)	200 (120–340)	200
RT-ddPCR	47 (28–79)	200 (120–340)	200

### Comparison of Precision Between RT-qPCR and RT-ddPCR

The overall SD (i.e. residual SD from linear regression analyses, from log_10_ transformed data) from LOQ up to the neat dilution level was 0.14 with RT-qPCR and 0.20 with RT-ddPCR. The difference in precision between the two methods was significant (*p* = 0.031).

### Quantitative Agreement Between RT-qPCR and RT-ddPCR

**Fig. 2:**
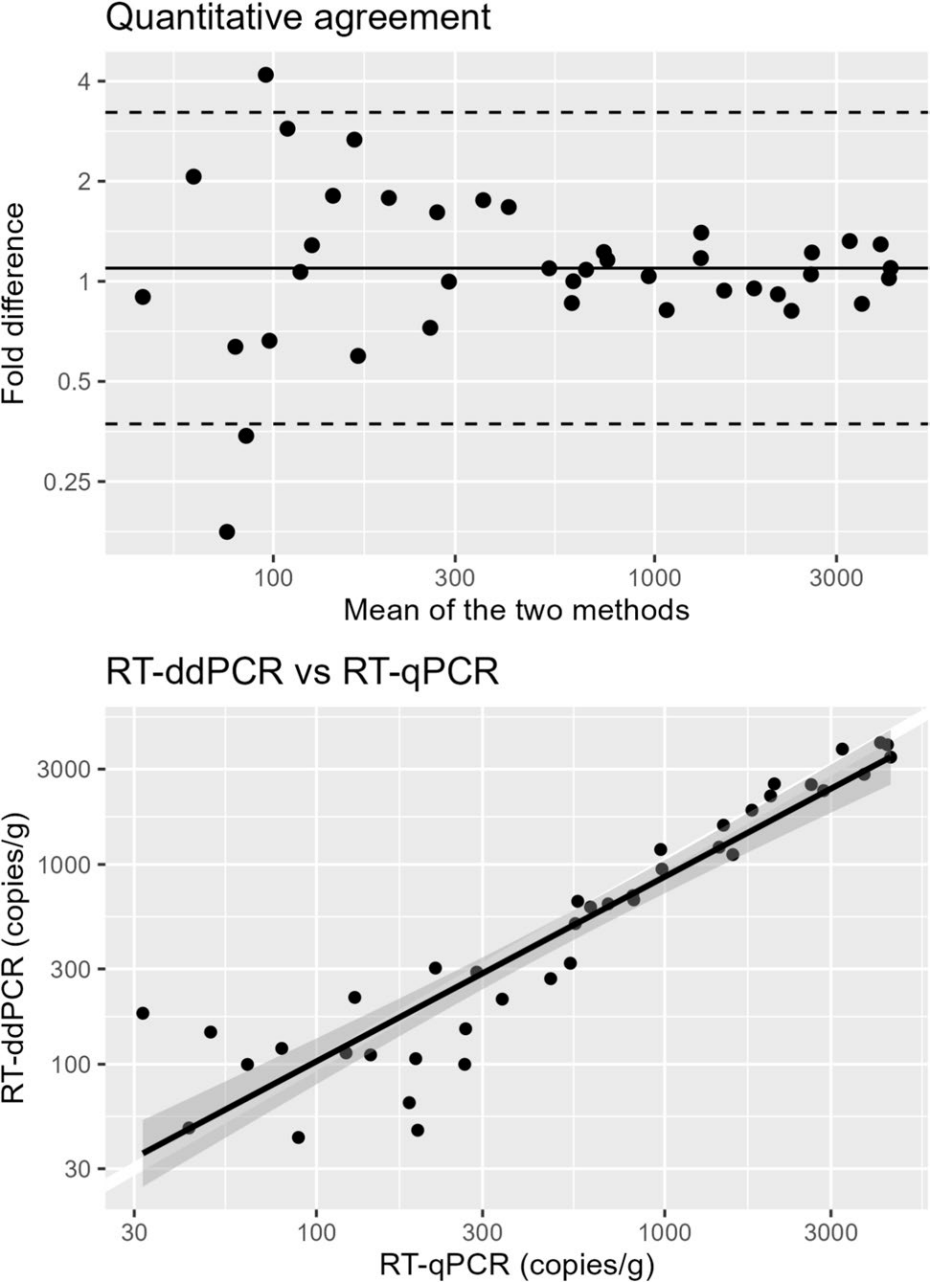
Top: Bland and Altman diagram displaying quantitative agreement between RT-qPCR and RT-ddPCR. The x-axis indicates the mean of the two measurements and the y-axis indicates the difference of the two measurements. Dots represent observed values, the thick horizontal line indicates the mean difference between the methods (estimated at 1.1, meaning that the RT-qPCR concentration was on average 10% higher than the RT-ddPCR concentration), and the dashed lines indicate the limits of agreement (mean ± 2 SD; 0.37–3.2). Bottom: estimated concentration with RT-ddPCR versus RT-qPCR. Dots represent observed values. The thick black line represents fitted values from linear regression the shaded areas represent a pointwise 95% confidence intervals of the fitted values

RT-qPCR and RT-ddPCR agreed very well in quantification: the obtained RT-qPCR concentration was on average 1.1 times (i.e. 10%) higher than the obtained RT-ddPCR concentration (Fig. 2).

## Method Comparison

The protocols used in the method characterisation study (RNA concentration with isopropanol precipitation and RT-qPCR with Mastermix A and RT-ddPCR) were compared with other protocols. A total of six combinations were evaluated: isopropanol precipitation or Minimag together with RT-qPCR Mastermix A and B, and RT-ddPCR.

**Fig. 3:**
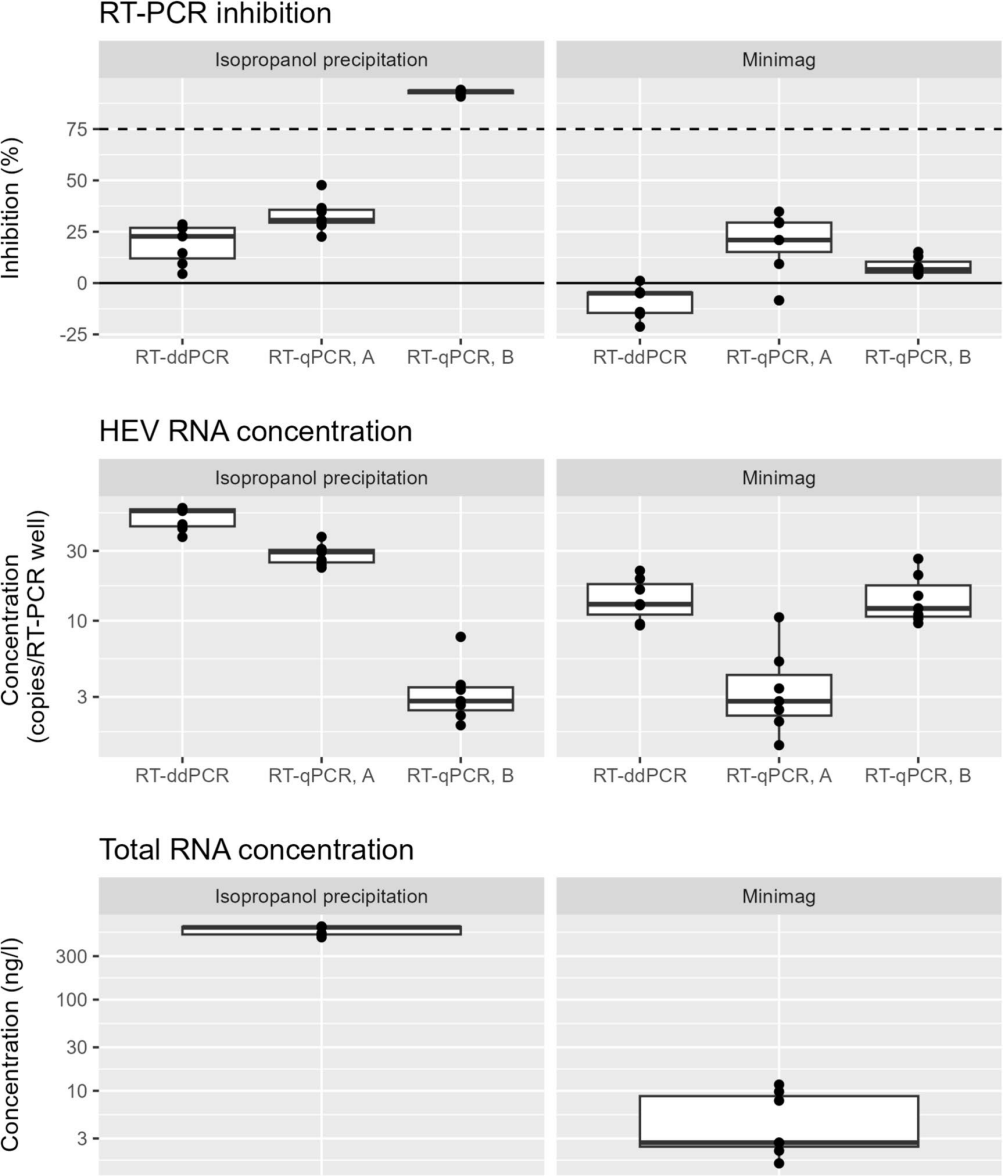
Comparison between different RNA purification methods and RT-PCR detection methods. Top: total RNA concentration. Middle: % inhibition in RT-PCR. The solid horizontal line indicates no inhibition and the dashed horizontal line indicates the maximum tolerable inhibition. Bottom: obtained concentration in RT-PCR. Mastermix A: TaqPath 1-Step RT-qPCR Master Mix, CG, Mastermix B: RNA UltraSense One-Step Quantitative RT-PCR System. Dots represent observed values, the boxes represent 50% of observations, the whiskers each represent 25% of observations (excluding outliers), and the thick horizontal lines represent the median

In terms of total RNA extraction yields, isopropanol precipitation yielded a higher concentration than Minimag extraction (*p* = 0.016) (Fig. 3, top). Regarding RT-PCR inhibition, all protocols except the combination isopropanol precipitation/RT-qPCR Mastermix B showed an acceptable value (≤ 75%, (Fig. 3, middle). With regard to the HEV concentration obtained in RT-PCR, the values were dependent on the combination of RNA purification/RT-PCR protocol (Fig. 3, bottom). Overall, the protocols used for characterisation, i.e. isopropanol precipitation with RT-qPCR Mastermix A and RT-ddPCR yielded the highest HEV concentrations, with no significant difference between the two (*p* = 0.11). These combinations yielded from 2- to 16-fold higher HEV concentrations than the other combinations, with corresponding *p* values from 0.025 to < 0.0001.

### In silico Inclusivity Analysis of HEV Primers and Probe

**Fig. 4:**
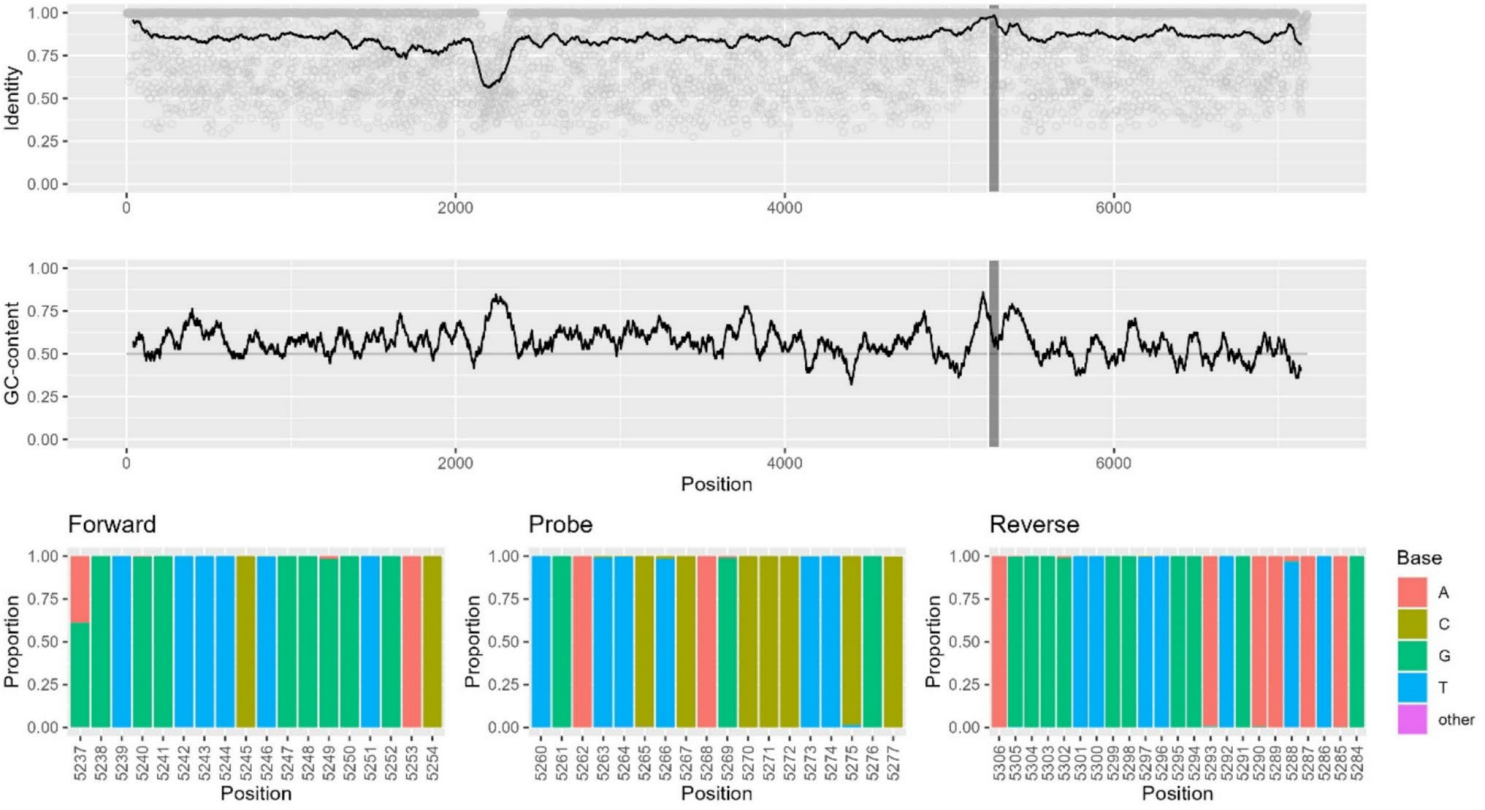
Sequence conservation analysis of the Orthohepevirus A genome and investigation of the binding regions of the primers and probe used in this study. Top: overview of the HEV genome with the RT-PCR assay location indicated. Dots represent the value at each position, horizontal lines represent running averages, and the vertical shaded area indicates the assay-binding region. Identity: proportion of the most frequently occurring base. GC-content: proportion of G or C. Bottom: nucleotide distribution within the primer and probe binding regions of the RT-PCR assay, shown in 5′–3′ direction of the oligos

To investigate how well the selected primers and probe matched their HEV target sequences, an i*n silico* analysis was performed with 834 *Orthohepevirus* A genomes of genotypes 1–7 (no genotype 8 genome was found at the time of the study, October 2022). Most of the sequences in the dataset belonged to genotype 3 (*n* = 490) and 4 (*n* = 114). The RT-PCR assay covered position 5237–5306 (with reference to NC_001434), which was the most conserved region of the otherwise highly variable genome (Fig. 4, top). The inclusivity was very good: the forward primer matched perfectly to 59% of the sequences (41% of the sequences had only one mismatch), the probe to 94%, and the reverse primer to 93% (Fig. 4, bottom). No sequence had more than two mismatches with each oligo.

### Evaluation of the RT-PCR Assay Using the WHO International Standard and Reference Panel for HEV RNA

Laboratory evaluation of the WHO International Standard (Baylis et al., 2013) using RT-ddPCR demonstrated that the quantification closely aligned with the expected HEV concentration. The intended concentration of the standard was 250,000 international units/ml, while RT-ddPCR measured it at 256,000 copies/ml. Given this high level of agreement, we used RT-ddPCR values directly to quantify the WHO International Reference Panel for HEV RNA.

**Fig. 5:**
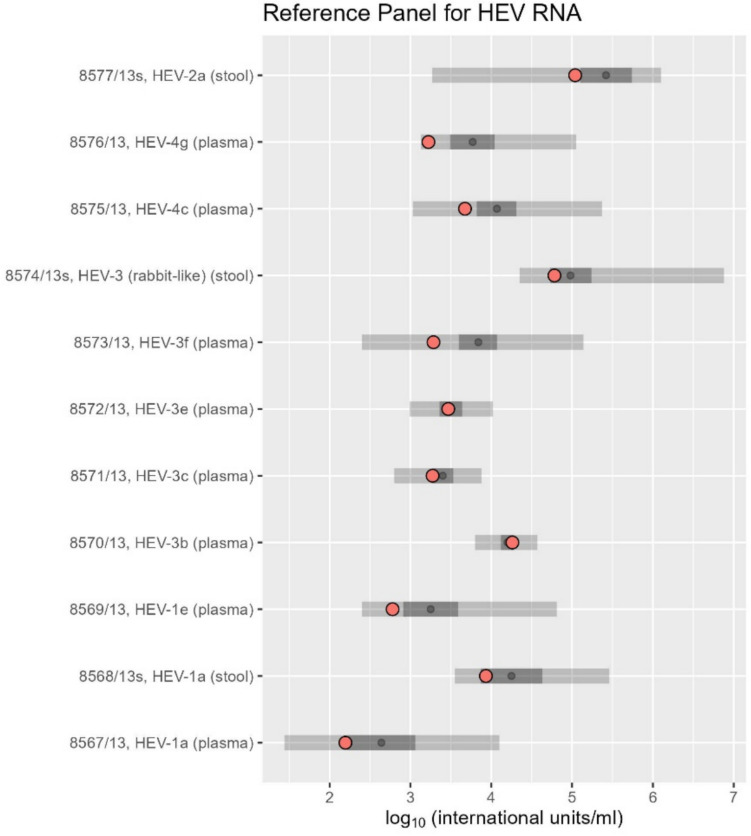
Results from the analysis of the WHO International Reference Panel for HEV RNA. The y-axis labels indicate the reference number, genotype and sub-genotype, and the source of each standard. Black dots represent the mean values obtained from a previous international collaborative study involving 17 laboratories performing quantitative HEV assays (Baylis et al., 2015), while the dark grey area denotes the 95% confidence interval (CI) of the mean. The light grey area indicates the full range of values from the collaborative study, and red dots represent the RT-ddPCR values obtained in this study. Given the near-perfect agreement between RT-ddPCR quantification and the WHO International Standard for HEV RNA, RT-ddPCR copy numbers per ml were used interchangeably with international units

No reference concentrations were provided with the Reference Panel. However, all 11 panel samples fell within the range of values reported in a previous international collaborative study involving 17 laboratories performing quantitative HEV assays (Baylis et al., 2015), confirming that the assay is able to detect and quantify important HEV genotypes and sub-genotypes (Fig. 5). However, our results were overall slightly lower than the mean values from the collaborative study, with an average deviation of − 0.31 log_10_ units (0.49 fold). The average inhibition was 7.7%, ranging from − 16% to 40%.

## Discussion

Over the past 10–15 years, the awareness of zoonotic HEV infections has been growing in Europe (Hazards et al., 2017). Contaminated sausages and similar products derived from domestic pigs or wild boars have been identified as important sources of infection in humans. To reduce the risk of HEV infection, the development of standardised and validated protocols for the rapid and efficient detection and quantification of HEV in such products is important. Several protocols for HEV detection in meat products have already been described [see for example: (Althof et al., 2019; Bartolo et al., 2012; Colson et al., 2010; Hennechart-Collette et al., 2019; Martin-Latil et al., 2014; Mykytczuk et al., 2017; Szabo et al., 2015)]. These protocols usually involve three steps: (1) homogenisation of the sample and release of viral particles/RNA, (2) extraction/concentration of viral RNA, and (3) molecular detection of HEV RNA, mainly by RT-qPCR.

For sample homogenisation, previously published protocols report the usage of a vortex (Szabo et al., 2015), Stomacher-type laboratory blender (Althof et al., 2019; Hennechart-Collette et al., 2019; Martin-Latil et al., 2014; Szabo et al., 2015), FastPrep machine (Hennechart-Collette et al., 2019; Szabo et al., 2015), or a mortar followed by zirconia-bead disruption (Bartolo et al., 2012). Prior to this study, we evaluated homogenisation using both a Stomacher device and an IKA ULTRA THURRAX disperser. We could not find any major difference between the two strategies in terms of total RNA, HEV, and mengovirus concentrations (Table S1 in Supplementary Material), and therefore the IKA ULTRA THURRAX system was selected because it can be conveniently used in a laminar flow hood system. Similar to a previously published protocol (Mykytczuk et al., 2017), we chose to use tri-reagent solution (a mixture of phenol and guanidine thiocyanate) during homogenisation in combination with 1-bromo-3-chloropropane to promote separation between the protein, DNA, and RNA phases.

For extraction and concentration of viral RNA, guanidine thiocyanate-magnetic silica bead-based methods (such as Minimag) are widely used in food virology applications, including in protocols that utilise tri-reagent solution prior to the RNA concentration step (Althof et al., 2019; Hennechart-Collette et al., 2019; Martin-Latil et al., 2014; Szabo et al., 2015). Commercial spin kits (QIAamp Viral RNA Mini Kit from Qiagen) (Bartolo et al., 2012) and extraction robots (EZ1 VirusMini kit with BioRobot EZ1 from Qiagen) (Colson et al., 2010) are also used. We chose a traditional isopropanol precipitation protocol for RNA concentration (Mykytczuk et al., 2017) and could see several advantages with this approach: the procedure is inexpensive, fast, and requires no advanced or proprietary instrumentation.

To identify suitable primer and probe sets for HEV detection via RT-PCR, we conducted an extensive literature search and evaluated several published assays (Enouf et al., 2006; Garson et al., 2012; Gyarmati et al., 2007; Jothikumar et al., 2006; Martin-Latil et al., 2012, 2014). Using our in silico inclusivity assessment method, we selected a system originally developed by Jothikumar et al. (2006) and later modified by Garson et al. (2012) and Martin-Latil et al. (2012). The system demonstrated good PCR efficiency (Table S2 in Supplementary Material), and its performance was verified through the analysis of the WHO International Reference Panel for HEV RNA (Fig. 5). Although these primers and probes had been previously described, we found that the inclusivity analysis was a valuable screening tool for identifying the most suitable candidates. Additionally, the significant increase in the number of available HEV genomes on GenBank since the publication of these assays emphasises the importance of updated analyses.

For characterisation of the method, we employed an already-established procedure, previously used to validate ISO 15216–1 (Lowther et al., 2019), with minor modifications. Of the previously published protocols mentioned in this paper (Althof et al., 2019; Bartolo et al., 2012; Colson et al., 2010; Hennechart-Collette et al., 2019; Martin-Latil et al., 2014; Mykytczuk et al., 2017; Szabo et al., 2015), two report data on LOD for the detection of HEV in pork sausages (Martin-Latil et al., 2014; Szabo et al., 2015). However, our results (Table 3) cannot be directly compared to theirs results for several reasons. The different studies use different types of quantification standards, sausage, protocols, and approaches for the LOD calculation and for determining the anticipated concentrations. We used frankfurter sausage as a model and found that LOD_95%_ and LOQ were 200 copies/g. Szabo and colleagues (Szabo et al., 2015) assessed LOD in raw and pork liver sausage artificially contaminated with four contamination levels of HEV, with triplicate samples for each level. In their study, LOD was 580 copies/g in raw sausage and 26,500 copies/g in liver sausage. In addition, Martin-Latil and colleagues (Martin-Latil et al., 2014) assessed LOD in figatelli and pork liver sausage artificially contaminated with five contamination levels of HEV, with eight samples for each level. In their study, LOD was 2900 copies/g in figatelli and 29,000 copies/g in pork liver sausage. None of the above studies provides data on e.g. LOQ or precision. However, the method described by Szabo and colleagues (Szabo et al., 2015) has been further evaluated in an interlaboratory comparison for qualitative detection (Althof et al., 2019).

Mengovirus was selected as a positive process control because it is widely used and proposed for use as such in ISO 15216–1 (I.S.O., 2017). The mengovirus and HEV extraction efficiencies were in close agreement, but the average values were above 100% for both targets. This indicates that the tri-reagent-based protocol for sausage samples provides much more efficient RNA extraction than the boil lysate procedure for positive extraction control samples. In the method development phase of this study, we first attempted to extract the positive extraction control using tri-reagent solution and 1-bromo-3-chloropropane, followed by isopropanol precipitation (i.e. we used a similar protocol as for the sausage samples but adapted it to a smaller sample volume), but the tri-reagent-based protocol proved to be highly inefficient for positive extraction control samples. We also compared a boil lysate positive extraction control with a Minimag positive extraction control and found that the obtained concentration of the Minimag positive extraction control appeared to be slightly lower compared to that from the boil lysate positive extraction control (Table S3 in Supplementary Material).

According to ISO 15216–1:2017, a sample is valid if the extraction efficiency is at least 1% (I.S.O., 2017). However, given the high values observed for our method, a threshold of 1% is probably too low. Approaches to define appropriate quality criteria for the minimum extraction efficiency therefore remain subject for further investigation.

Although HEV is an RNA virus, we chose to use DNA standards for quantification in RT-qPCR. This decision was primarily made to align with the quantification procedures outlined in ISO 15216–1:2017. Furthermore, we aimed to harmonise the quantification process with RT-ddPCR, which quantifies cDNA copies rather than RNA. DNA standards also offer greater stability over time and are easier to handle from a practical standpoint. Importantly, we found overall good agreement between quantification results obtained with RT-qPCR and RT-ddPCR (Fig. 2), and RT-ddPCR quantification was essentially identical to the WHO International Standard for HEV RNA quantification (Baylis et al., 2013). Consequently, both RT-qPCR and RT-ddPCR results from this study can likely be used almost interchangeably with the WHO international units for HEV RNA quantification.

While RT-qPCR is currently the most widely used technique for detecting viruses in food, digital PCR has emerged as a promising alternative. The proposed advantages include improved precision in quantification and increased accuracy as no external quantification standards are required (Hindson et al., 2013). Digital PCR-based methods have previously been used to quantify HEV in human plasma, pork liver, and retail pork products (Martin-Latil et al., 2014, 2016; Mykytczuk et al., 2017; Nicot et al., 2016). However, none of these studies report performance data on LOD and LOQ compared to RT-qPCR. Considering that RT-ddPCR is generally more expensive and time-consuming than RT-qPCR, we found no support for using RT-ddPCR over RT-qPCR for this method. Also, an important observation was that positive RT-ddPCR signals were found in non-spiked sausage, NPC and NTC samples. Such signals are problematic because they affect the ability of the method to accurately detect low target concentrations. Although we have not applied one here, a cut-off value (limit of blank) may be needed in the future to safely distinguish between true- and false-positive signals in RT-ddPCR.

After performing the method characterisation, we compared different combinations of RNA purification methods and RT-PCR protocols. For RNA purification, we used isopropanol precipitation and Minimag extraction, and for RT-PCR, we used RT-qPCR with two different master mixes (A: TaqPath 1-Step RT -qPCR Master Mix, CG, B: RNA UltraSense One-Step Quantitative RT-PCR System) and RT-ddPCR. We chose to compare our validated protocols with Minimag extraction and RT-qPCR with mastermix B because it is widely used in food virology and is included in an informative annex in ISO 15216–1:2017 (I.S.O., 2017).

The isopropanol protocol was found to yield substantially higher total RNA concentrations compared to the Minimag protocol. However, the differences in HEV concentration determined with RT-PCR were not nearly as pronounced, and the results were highly dependent on the specific *combination* of RNA purification and RT-PCR protocol (Fig. 3). One possible explanation for this is that while the isopropanol protocol may provide the highest total RNA yield, the magnetic silica beads in the Minimag extraction procedure may bind more selectively to the target RNA while avoiding impurities that could potentially interfere with the RT-PCR reaction and thereby provide less RT-PCR inhibition. It is important to find a balance as excessive purification of samples can lead to loss of target RNA. Here, the use of an inhibitor-tolerant RT-PCR mastermix such as Mastermix A after isopropanol precipitation offers a pragmatic solution that can eliminate the need for additional purification steps (Hedman et al., 2013; Rådström et al., 2004).

A limitation of this study is the lack of naturally infected food samples. We artificially contaminated the food samples by adding HEV particles to the samples followed by mixing. However, HEV can also be present inside cells in naturally infected pork products. Thus, for this method to work properly, the cells must be efficiently lysed to release viral particles and RNA before the RT-PCR analysis. Naturally contaminated sausages were unfortunately not available to us at the time of the study, but we successfully used the method to detect HEV in wild boar liver previously tested as HEV positive by the Swedish Veterinary Institute (Table S4 in Supplementary Material).

In conclusion, our study presents a characterised method for the quantitative detection of HEV in pork sausage. Robust methods with well-defined performance parameters are crucial for reliable data generation in future risk assessments and surveillance studies.

## Supplementary Information

Below is the link to the electronic supplementary material.

Supplementary file1 (DOCX 264 KB)

## Data Availability

All datasets, analysis code, and raw data related to this article are available upon request to the corresponding author.
