# Supplementary material for: Validation of an Optimised Method for Quantitative Detection of Hepatitis E Virus in Pork Sausage
Source: Food Environ Virol. 2025 Jun 2;17(2):33. doi: 10.1007/s12560-025-09645-3 (PMC12130118; doi:10.1007/s12560-025-09645-3)
Supplement: Supplementary file 1 — Supplementary file1 (DOCX 264 KB) [file 12560_2025_9645_MOESM1_ESM.docx]

Validation of an optimised method for quantitative detection of hepatitis E virus in pork sausage

Sofia Persson^a,b*^, Ramia Molin^a^, Ronnie Eriksson^a^, Moa Lavander^a^, Frederik Widén^c^, Patrik Ellström^b^, Magnus Simonsson^a^

^a^European Union Reference Laboratory for Foodborne Viruses, Swedish Food Agency, Dag Hammarskjölds väg 56 A, 752 37, Uppsala, Sweden

^b^Department of Medical Sciences, Zoonosis Science Centre, Uppsala University, Husargatan 3, 752 37, Uppsala, Sweden

^c^Department of Microbiology, Swedish Veterinary Institute, Ulls väg 2B, 751 89 Uppsala, Uppsala, Sweden

^*^Corresponding author, email: [sofia.persson@medsci.uu.se](mailto:sofia.persson@medsci.uu.se)

**Table S1.** Comparison between homogenisation using an IKA ULTRA THURRAX and Stomacher apparatus with two different sausage types artificially contaminated with the same amount of HEV

| Sample | Food type | Homogenisation method | Obtained HEV concentration (copies/g) | Obtained mengovirus concentration (units/sample) | Nanodrop total RNA concentration (ng/g) | Nanodrop A260/280 |
| --- | --- | --- | --- | --- | --- | --- |
| 1 | Frankfurter | IKA | 7,22E+03 | 8,21E+05 | 4.93E+04 | 1.95 |
| 2 | Frankfurter | IKA | 9,17E+03 | 1,27E+06 | 7.14E+04 | 1.96 |
| 3 | Salami | IKA | 2,93E+03 | 4,61E+05 | 6.67E+04 | 1.90 |
| 4 | Salami | IKA | 2,98E+03 | 3,94E+05 | 4.67E+04 | 1.88 |
| 5 | Frankfurter | Stomacher | 7,38E+03 | 1,25E+06 | 5.90E+04 | 1.95 |
| 6 | Frankfurter | Stomacher | 1,03E+04 | 1,32E+06 | 6.91E+04 | 1.97 |
| 7 | Salami | Stomacher | 4,34E+03 | 8,34E+05 | 2.22E+04 | 1.87 |
| 8 | Salami | Stomacher | 3,92E+03 | 6,23E+05 | 3.50E+04 | 1.87 |

**Table S2.** Performance of the HEV RT-qPCR assay, as estimated by 15 runs of a HEV DNA standard curve. The standards were run as described in Section 2.1.4.4 in the main article

| Run | Intercept | Slope | *R^2^* | Efficiency (%) |
| --- | --- | --- | --- | --- |
| 1 | 38.12 | -3.46 | 1.00 | 94.64 |
| 2 | 35.89 | -3.11 | 0.99 | 109.80 |
| 3 | 37.63 | -3.47 | 1.00 | 94.32 |
| 4 | 38.14 | -3.53 | 1.00 | 92.05 |
| 5 | 38.71 | -3.49 | 1.00 | 93.47 |
| 6 | 37.70 | -3.38 | 1.00 | 97.79 |
| 7 | 38.45 | -3.54 | 1.00 | 91.82 |
| 8 | 37.91 | -3.47 | 1.00 | 94.14 |
| 9 | 37.65 | -3.43 | 1.00 | 95.68 |
| 10 | 37.53 | -3.34 | 1.00 | 99.12 |
| 11 | 37.28 | -3.40 | 1.00 | 97.04 |
| 12 | 38.34 | -3.41 | 1.00 | 96.47 |
| 13 | 37.60 | -3.46 | 1.00 | 94.67 |
| 14 | 37.32 | -3.28 | 1.00 | 101.63 |
| 15 | 39.22 | -3.59 | 1.00 | 89.91 |
| Mean | 37.83 | -3.42 | 1.00 | 96.17 |

**Table S3.** Comparison boil lysate and Minimag PEC for HEV and mengovirus. The PECs were run as described in Section 2.1.3 in the main article

| Type of control | HEV, Cq | HEV, concentration (copies/PEC sample) | Mengovirus, Cq | Mengovirus, concentration (units/PEC sample) |
| --- | --- | --- | --- | --- |
| Boil lysate | 28.63 | 1.34E+04 | 17.95 | 5.16E+04 |
| Minimag | 29.08 | 1.78E+04 | 19.65 | 1.71E+04 |

**Table S4.** Detection of HEV in wild-boar liver using a modified version of the sausage protocol that was validated in the main article (the protocol was adjusted for smaller volumes, and detection was only performed using RT-qPCR). The samples were previously identified as HEV positive by qualitative RT-PCR analysis performed at the Swedish Veterinary Agency (SVA)

| Sample number | Analysed amount (g) | Cq value | HEV concentration (copies/g) |
| --- | --- | --- | --- |
| 9020 | 0.40 | 17.25 | 2.75E+08 |
| 1712 | 0.40 | 34.56 | 1.60E+03 |
| 141 | 0.40 | 37.21 | 4.03E+02 |


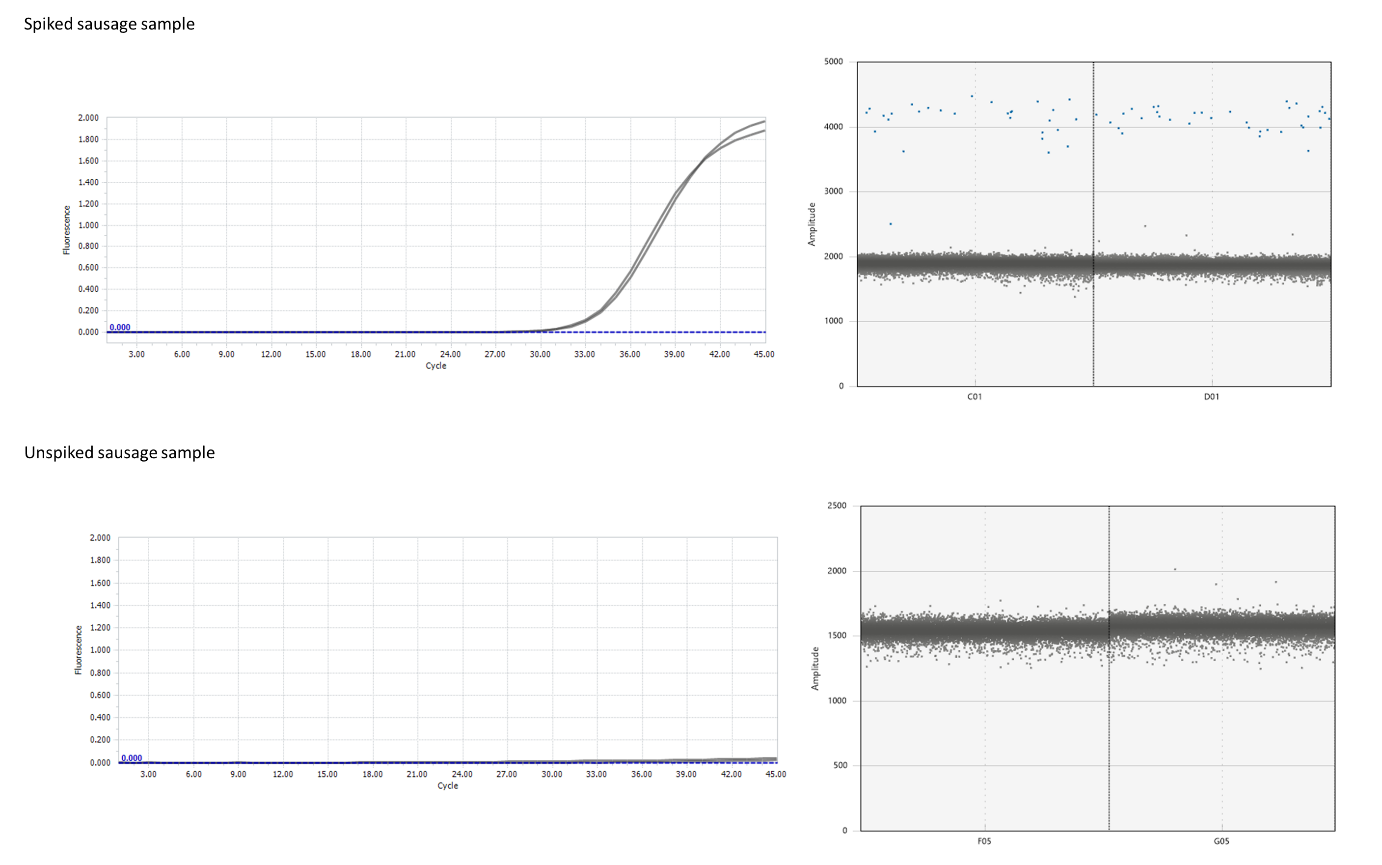


**Figure S1.** Performance of the HEV assay in RT-qPCR and RT-ddPCR, exemplified by two replicate wells of a spiked and unspiked sausage sample.
